# Short-term responses of *Rana arvalis* tadpoles to pH and predator stress: adaptive divergence in behavioural and physiological plasticity?

**DOI:** 10.1007/s00360-022-01449-2

**Published:** 2022-07-20

**Authors:** Nicholas Scaramella, Jelena Mausbach, Anssi Laurila, Sarah Stednitz, Katja Räsänen

**Affiliations:** 1 Animal Ecology/Department of Ecology and Genetics, Evolutionary Biology Centre, Uppsala University, Norbyvägen 18D, 75236 Uppsala, Sweden; 2 Department of Aquatic Ecology, Eawag, Ueberlandstrasse 133, 8600 Duebendorf, Switzerland; 3 Institute of Integrative Biology, ETH Zurich, Universitätstrasse 16, 8092 Zurich, Switzerland; 4 Department Sensory and Sensorimotor Systems, Max Planck Institute for Biological Cybernetics, Max-Planck-Ring 8, 72076 Tübingen, Germany; 5 Present Address: Department of Biological and Environmental Science, University of Jyväskylä, Survontie 9C, 40014 Jyväskylä, Finland; 6 Present Address: Department of Ecology, Swedish University of Agricultural Sciences, Almas Alé 8, 75007 Uppsala, Sweden

**Keywords:** Adaptive divergence, Behaviour, Corticosterone, Phenotypic plasticity, *Rana arvalis*

## Abstract

Environmental stress is a major driver of ecological and evolutionary processes in nature. To cope with stress, organisms can adjust through phenotypic plasticity and/or adapt through genetic change. Here, we compared short-term behavioural (activity) and physiological (corticosterone levels, CORT) responses of *Rana arvalis* tadpoles from two divergent populations (acid origin, AOP, versus neutral origin, NOP) to acid and predator stress. Tadpoles were initially reared in benign conditions at pH 7 and then exposed to a combination of two pH (acid versus neutral) and two predator cue (predator cue versus no predator cue) treatments. We assessed behavioural activity within the first 15 min, and tissue CORT within 8 and 24 h of stress exposure. Both AOP and NOP tadpoles reduced their activity in acidic pH, but the response to the predator cue differed between the populations: AOP tadpoles increased whereas NOP tadpoles decreased their activity. The AOP and NOP tadpoles differed also in their CORT responses, with AOP being more responsive (CORT levels of NOP tadpoles did not differ statistically across treatments). After 8 h exposure, AOP tadpoles had elevated CORT levels in the acid-predator cue treatment and after 24 h exposure they had elevated CORT levels in all three stress treatments (relative to the benign neutral–no-cue treatment). These results suggest that adaptation to environmental acidification in *R. arvalis* is mediated, in part, via behavioural and hormonal plasticity.

**Supplementary Information:**

The online version contains supplementary material available at 10.1007/s00360-022-01449-2.

## Introduction

Environmental stress, defined as any outside influence that reduces organismal performance and fitness, is a powerful evolutionary force (Hoffmann and Parsons 1997; Hoffmann and Hercus 2000). When different populations inhabit contrasting stress environments, environmental stress can lead to strong divergent natural selection and facilitate local adaptation (Kawecki and Ebert 2004). An alternative, but not mutually exclusive, mechanism for within-generation stress responses arises via phenotypic plasticity (Pigliucci 2001; Ghalambor et al. 2007), which can help mitigate effects of environmental stressors (e.g. Relyea 2004; Auld et al. 2010; Merilä and Hendry 2014; Chevin and Hoffmann 2017; Fox et al. 2019; Norin and Metcalfe 2019). Importantly, environmental stressors typically covary in nature, resulting from simultaneous shifts of abiotic and biotic factors, which can lead to trade-offs in fitness related traits (Folt et al. 1999; Vinebrooke et al. 2004; Hopkins et al. 2017). As many environmental stressors fluctuate strongly in space and time, short-term stress responses can be important components and indicators of reversible phenotypic plasticity (Gabriel 2005), and are often measured as behavioural modifications (e.g. reduced activity or increased erratic behaviour) or altered levels of glucocorticoids (cortisol or corticosterone) (reviewed in Gormally and Romero 2020). However, to what extent populations differ in behavioural and physiological stress responses is poorly understood.

Physiologically stressful conditions, such as extreme temperature or pH, necessitate organismal investment to maintenance of physiological balance, whilst ensuring other key functions, such as resource acquisition and evasion from predators (e.g. Maltby 1999). Predator presence is an ubiquitous biotic stressor in nature. To avoid being eaten, prey often alter their behaviour and develop morphologically distinct defense traits (e.g. Lima and Dill 1990; Tollrian and Harvell 1999; Ferrari et al. 2010). Plastic alteration of activity level is one of the most prevalent responses, whereby prey typically reduces activity to avoid detection (Lima and Dill 1990; Kats and Dill 1998). While such behavioural responses increase the immediate survival chances of the prey, they can have negative long-term consequences (e.g. reduced growth, development or reproduction; Tollrian and Harvell 1999; Ferrari et al. 2010).

Aquatic larval stages of many amphibians are well-suited to study plastic and genetic responses to stress as they are sensitive to environmental change, occur in distinct environments and often show strong local adaptation (Beebee 2005). In amphibians, stressors such as predators activate the neuroendocrine stress axis (hypothalamic–-pituitary–interrenal axis, HPI), with corticosterone (henceforth CORT; Denver 2009) as the main hormonal mediator of stress responses. In the short term, elevated CORT levels can allow rapid energy mobilization to different stress responses (Denver 2009). However, maintaining high CORT levels over extended periods can come at the cost of reduced growth or delayed development (Denver 2009; Middlemis Maher et al. 2013). Hence, the ability to plastically modify CORT levels in response to short-term stress can be favoured in spatially and temporally heterogeneous environments (e. g. Burraco et al. 2013; Narayan et al. 2019; Vitousek et al. 2019), but without the ability to reliably detect these stressors the plastic traits would be of only limited advantage.

In aquatic systems, prey often detect predators via chemical cues that are emitted both by predators, as well as by the prey itself when attacked (Kats and Dill 1998; Schoeppner and Relyea 2005; Ferrari et al. 2010; Van Buskirk et al. 2014). However, if environmental conditions impair their chemosensory responses prey may not be able to detect these chemical cues, such as may be the case for aquatic organisms in acidic environments (reviewed in Leduc et al. 2013). Natural and human induced environmental acidification is a major source of multiple stressors (e.g. Egea-Serrano et al. 2014). On one hand, acidic pH can have strong negative physiological effects on a range of organisms (e.g. Schindler et al. 1985; Rusek and Marshall 2000), including amphibians (reviewed in Räsänen and Green 2009). On the other hand, acidification causes substantial changes in community structure, such as elevated densities of insect predators as fish disappear (Henrikson 1990). As acid stress reduces growth and development rates as well as survival of amphibians (reviewed in Räsänen and Green 2009), and predator stress causes behavioural and morphological alterations and reduces survival, these strong fitness consequences of acidification can lead to multidimensional divergent selection (Egea-Serrano et al. 2014). Physiological stress responses of tadpoles to acidity are evident typically as reduced behavioural activity (Freda and Taylor 1992; Räsänen et al. 2002; Egea-Serrano et al. 2014) and disrupted ion balance (Freda and Dunson 1984; Meyer et al. 2020), and both predators and acidity can alter CORT expression (e.gChambers et al. 2013; Florencio et al. 2020). Furthermore, behavioural and physiological traits can be tightly linked, with some studies showing a positive correlation between behavioural activity and CORT expression (Cote et al. 2006; Cottin et al. 2011). Immediate responses, such as behavioural responses to predator cues and physiological acclimation to pH, can play a major role in coping with environmental acidification and be under divergent selection.

In southwestern Sweden, moor frog (*Rana arvalis)* populations show adaptive divergence in multiple traits along an acidification gradient (e.g. Räsänen et al. 2003, 2008; Hangartner et al. 2012a, b; Egea-Serrano et al. 2014; Shu et al. 2015)—reflecting responses to divergent selection via predators and pH. However, short-term behavioural and physiological responses to acidity and predator stress, and potential population level divergence in short-term stress responses (i.e. genotype–environment interactions, Pigliucci 2001), remain unknown. Here we studied short-term behavioural and CORT responses of *R. arvalis* tadpoles to biotic (predator cue) and abiotic (acidic pH) stress interactions in two populations that represent opposing ends of a pH gradient (breeding pond pH 4, henceforth AOP, and pH 7, henceforth NOP), and show substantial behavioural, morphological and life-history divergence: AOP tadpoles are more active, have deeper tails, are larger and have higher survival than NOP tadpoles when exposed chronically to acid and predator stress (e.g. Hangartner et al. 2012a, b; Egea-Serrano et al. 2014).

To study short-term stress responses, we reared tadpoles in the laboratory initially under benign conditions (pH 7.5, no predator cues). At mid-larval stages, the tadpoles were exposed to a combination of two pH (neutral versus acidic pH) and two predator (no cue versus predator cue) treatments and assessed for behavioural activity (before and shortly after addition of cue) and tissue corticosterone (CORT) levels (after 8 or 24 h). We predicted that if acidic water impairs the detection of predator cue (Leduc et al. 2013), tadpoles would show a reduced behavioural or CORT response in the acid-predator cue treatment relative to neutral-predator cue treatment. However, due to different pH and predator induced selective histories of the two populations, we further expected different responses between AOP and NOP tadpoles.

## Materials and methods

### Study system

*Rana arvalis* inhabits a broad range of aquatic habitats and acidity levels throughout much of central and northern Europe (Glandt 2006). It breeds in early spring shortly after snow melt and the aquatic tadpoles develop to metamorphosis in ca. 2–3 months (Glandt 2006). Two previously studied populations in southwestern Sweden (Hangartner et al. 2011) were chosen for the study: Tottatjärn (breeding pond pH 4.0 ± 0.2; coordinates: 57°36′12"N 12°34′47"E) represents an acid location (henceforth AOP) and Rud (pH 7.0 ± 0.2; 58º35′28''N 13º47′26''E) a neutral location (henceforth NOP). These two populations situate ca. 130 km apart along an acidification gradient, where pH in breeding ponds differs due to natural and anthropogenic acidification and limestone bedrock (Hangartner et al. 2011). Earlier studies along the acidification gradient found a negative correlation between pond pH and the abundance of insect predators (Hangartner et al. 2011), providing evidence that invertebrate predators are more prominent in acidic ponds (Henriksson 1990).

### Egg collection and animal maintenance

Between 17 and 20th of April 2018, roughly 100 fertilized eggs were collected from a total of 10 clutches (i.e. full-sib families) per population. To minimize bias due to environmental effects, all embryos were collected within 1 h from egg-laying (at ca. 2-cell stage), and placed in clutch specific groups in plastic containers containing pH 7.5 reconstituted soft water (RSW, Räsänen et al. 2003). The embryos were maintained cool (at 4–7 °C) and transported to a climate-controlled laboratory at Uppsala University. At the lab, the embryos were reared in 0.8L of RSW (pH 7.5) in 1L PP containers (11 × 11 × 12 cm). The embryos were reared in family specific groups (ca. 40–50 embryos/container) until they hatched, and larvae started independent feeding (stage G25; Gosner 1960). Water was changed every 2–3 days to maintain good water quality.

At stage G25, the tadpoles were randomly assigned to individual 1L experimental containers (see below), equipped with a folded piece of non-transparent plastic as shelter. The tadpoles were fed ad libitum with a mixture of spinach and spirulina (200 g frozen spinach and 8.08 g dried spirulina in 10 ml of RSW). Each individual was fed every third day, in conjunction with water change, and the amount of food was increased as the animals grew. By stage G32 (Gosner 1960), the mid-larval stage studied here, the tadpoles received roughly 0.5 ml of food mixture per feeding. The health of the tadpoles was checked during each water change. Two tadpoles died shortly after set-up and two other individuals were humanely sacrificed (their development was stagnated, or they showed signs of discomfort or neurological abnormalities (e.g. swimming in circles)). As tadpoles in this experiment were only exposed to different treatments at the end of the experiment, these four individuals were replaced by ‘extra individuals’ that had been reared under similar conditions.

Individuals were reared in randomly assigned fixed spots on experimental shelving units in a walk-in acclimated room at 17 °C and 17L: 7D light cycle. To minimize errors in treatments and individual identification during the experiment, all people handling tadpoles had access to an ID list with respective population-treatment combinations. The average pH and temperature was measured from a subset of rearing containers using a Ross ultra-refillable triode with a portable pH meter Orion 3star pH (Thermo Scientific) and a digital thermometer (Testo 108, EN 13,485, ± 0.5 °C), respectively. Average pH was 7.61 ± 0.04 and temperature 16.08 ± 0.05 °C.

### Experimental design and rearing

Throughout the experiment, ARRIVE guidelines were followed. All tadpoles were reared initially at benign conditions at pH 7.5 RSW and in the absence of predator cues until mid-larval stage. Once they reached an approximate mid-larval stage of G32 (27.5–33) (Gosner 1960), the experimental manipulations started. As our interest was in responses to short-term stress, we maintained all individuals at benign (pH 7.5, no predator cue) conditions before they were exposed to pH and/or predator stress. We chose pH 7.5 for both populations as our earlier studies show that *R. arvalis* tadpoles, including those from AOP populations, have higher survival, growth and developmental rates at pH 7 than at more acidic pHs—indicating that pH 7 is physiologically benign for this species (e.g.Hangartner et al. 2011; Mausbach et al. 2022). The experiment was conducted as a 2 × 2 × 2 factorial design, with two populations (AOP and NOP), two pH treatments (acid: target pH 4.2 and neutral: target pH 7.5) and two predator treatments (predator cue and no cue) and 20 replicate individuals (total *N* = 160). Two or three individuals from each of the nine families were used within each population-treatment combination.

**Fig. 1:**
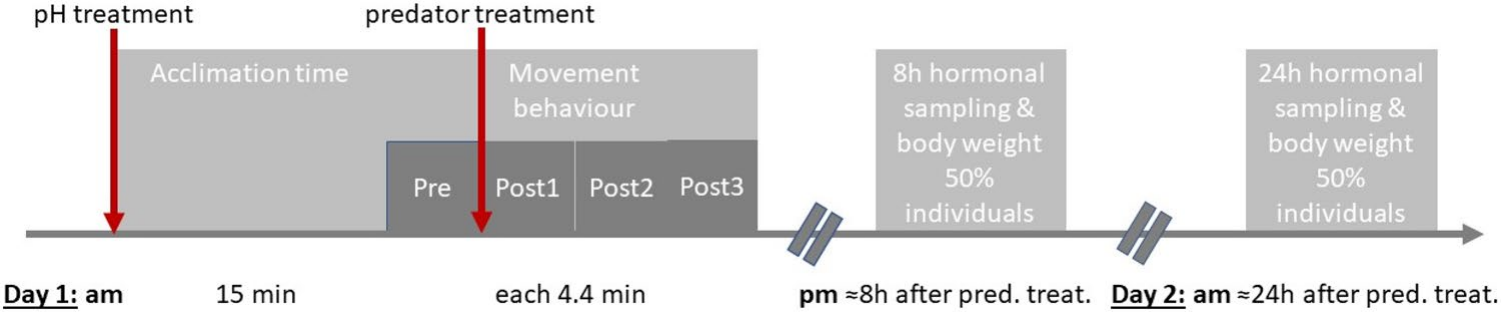
Experimental sampling procedure visualized. *Rana arvalis* tadpoles from each of the two study populations (AOP and NOP) were reared individually at physiologically benign pH (pH 7.5) from hatching until mid-larval stage. At approximate mid-larval stage (“G32”), individuals were exposed to four different treatment combinations (acid versus neutral water with or without predator cue). Day 1: Placement in acid (pH 4.2) or neutral (pH 7.5) water to acclimate for 15 min, followed by 5 min of video recording (i.e. Pre Time Point). After the 5 min recording, predator cue or no-cue control was added and video recording continued for an additional 15 min. Behaviour was measured from video´s at the following time points: **A** Pre: the 4.4 min before cue was added, **B** Post 1: the period immediately after cue was added (cue addition time = 0) to 4.4 min after cue addition, and **C** Post 2/3: 4.5–13.2 min after cue addition (i.e. Post2/2 consists of a 3 × 4.4 min period). After the recording was complete, half of the replicates (*N* = 10 for each population-treatment combination) were assigned for corticosterone sampling 8 h (Day 1) and the other half (*N* = 10 for each population-treatment combination) 24 h (Day 2) after the predator treatment was applied. For further details see methods

From each individual, we collected data on behavioural activity from video recordings and sampled whole-body CORT levels. For behavioural analyses, individuals were first exposed to the respective acid or neutral treatment and allowed to acclimate for 15 min before video recording commenced (see Fig. 1 for the sampling sequence). Individuals to be used within a given time point were decided based on a separate subset of ‘extra’ individuals that that were reared under similar conditions in the lab and had reached the approximate developmental stage of G32 (Mausbach 2021). This resulted in some variation in developmental stage in the sampled tadpoles (range G27.5 to 33). The NOP tadpoles also developed somewhat faster than the AOP tadpoles, and hence were sampled two days prior to the AOP tadpoles.

### Water preparation

Unmanipulated RSW has a pH of 7.2–7.6 and was prepared in 200 L Nalgene tanks and aerated. In the acid treatment (target pH 4.2), pH was lowered using 1 M H_2_SO_4_ and adjusted as needed for at least 2 days before being used in the experiments. In the acid pH treatment, 165 g peat pellets (Zoobest Gartenteich Torfpellets, ZB-01270) were added in a mesh bag to stabilize pH. In the neutral pH treatment, pH of the RSW was not adjusted, but 16.5 g peat pellets were added to control for the presence of humic compounds in the treatment water. The addition of peat is representative of humic compounds found in natural ponds in Sweden, with acidic ponds having higher levels than neutral ponds. During the experimental treatments, pH and temperature was not measured in the experimental containers as the exposure lasted a maximum of 24 h (pH is relatively stable over such short time; J. Mausbach, pers. obs.).

As predator cue, we used water collected from *Aeshna spp.* dragonfly larvae that had been fed with *R. arvalis* tadpoles. Predator cue water was prepared using 10 late-instar *Aeshna spp.* dragonfly larvae collected from a pond near Uppsala. Five individually caged *Aeshna* were kept in 80L of RSW (pH 7.5) within each of two 100L PP tanks. To collect the predator cue, each *Aeshna* (minimum length of 4 cm) was placed individually into a plastic container, with 100 ml water from their original holding tank, and fed roughly 120 mg of *R. arvalis* tadpoles. After 15 min, the *Aeshna* were returned to their holding tanks and water from the 10 cue collection containers was pooled. In the predator cue treatment, 2 ml of this water was pipetted to the tadpole container, which gave a concentration of roughly 3 mg tadpole/L of RSW in the tadpole vial (for details see Mausbach 2021). In the no-cue treatment, 2 ml of unmanipulated RSW (pH 7.5) was pipetted in the tadpole container as a technical control.

### Recording of behaviour

Activity of tadpoles was recorded (see Fig. 1) using three different video cameras simultaneously, with one camera recording 16 haphazardly selected individuals placed in individual containers (see below). Two sets of individuals were recorded per day. All recordings were done between 08:00 and 12:30 h to minimize variation due to circadian rhythm (e.g. Fraker 2008). Due to the differences in development time between the populations (see above), the video recordings of the NOP tadpoles were done two days before those of the AOP tadpoles (Fig. 1).

At the start of the behavioural trials, we selected three sets of 16 tadpoles haphazardly. These individuals were placed in their randomly assigned pH treatment (acid or neutral water) in 1L containers and transferred into a separate walk-in laboratory (18 °C), where the containers were placed randomly on a shelf in a 4 × 4 grid with a video camera (Sony HDR-CX250E) mounted 91 cm above the containers. The tadpoles were given a 15-min acclimation period after handling (no recording), followed by a 4.4 min (344 s) pre-cue recording (Fig. 1). Thereafter, predator cue or no-cue water was pipetted into the appropriate container and the activity of each tadpole recorded for 14.2 min (1032 s) (Fig. 1). To minimize disturbance, humans were not present during video recording except for turning the camera on and off, and adding the cue/control. Once video recording was completed, individuals were returned to the climate-controlled rearing room until hormonal sampling 8 or 24 h later (see below). Whilst video recordings were not conducted blind (to minimize errors in sampling), the analyses of the videos was conducted blind (by NS).

### Video analyses

Videos were analysed for behavioural activity using Image J2.0 with the Fiji extension, and Python (Version 3.7.2) with Canopy (Version 2.1.9.3717). All videos were cropped and converted to grayscale. A background image was generated by creating an average intensity Z projection of all frames. The background image was subtracted from the video (Stednitz et al. 2016) to highlight individual animal locations and increase tracking accuracy. Finally, all videos were thresholded to a binary image and eroded to minimise any single-pixel noise in the video. Using the ‘analyse particle’ function of ImageJ to detect objects in an image, the position of each animal was recorded in every frame, using a size threshold of four pixels (0.4 cm). Once all video recordings were processed, the video recording for each individual tadpole was separated into three packages reflecting time relative to exposure to predator cue (Cue addition = time 0). These were cut and standardized into Pre (the 4.4 min before cue was added), Post 1 (from the moment cue was added to 4.4 min) and Post 2/3 (from 4.4 to 13.2 min after cue was added) time points. (Note that for post 2/3 period, two 4.4 min periods were combined because preliminary analyses showed that tadpole behaviour did not differ between the Post 2 and Post 3 blocks; data not shown). The Pre-period reflects responses to pH treatments and represents behaviour prior to addition of predator cue (see above). Post1 and Post 2/3 represent the immediate and lag time (or recovery) in behavioural responses to predator cue treatments, respectively.

For statistical analyses, the percentage of time spent in motion per individual was calculated for each data period (Pre, Post 1 or Post 2/3) using custom code created in Python (by SS). We defined movement as any change in position greater than 5 pixels (0.5 cm) between frames in Python. To estimate percent movement for each individual, the number of incidents moved was divided by total of possible movement incidents (e.g. 500 movement incidents / 6600 total possible movement incidents = 0.075% movement). Validity of video analysis was confirmed through ICC analysis (Revelle 2020) compared to a manual count of a subset of videos (Supplemental Table 1). ICC analysis was also used to validate automatic movement tracking with Python. Both single fixed rater (ICC = 0.86, *p* < 0.001) and average fixed rater (ICC = 0.93, *p* < 0.001) analyses were highly correlated with manual tracking results (Cicchetti 1994).

### Hormonal sampling and measurement

For hormonal analyses, whole-body CORT samples of haphazardly chosen tadpoles were taken 8 h or 24 h after the stress treatments (predator cue and/or acid pH) started. In tadpoles, the first CORT response in the bloodstream is expected to be detectable after 5 min, whereas the distribution of CORT to tissues can take from 30 min to several hours, and the shape of temporal hormonal response curves can vary between developmental stages and species (e.g.Glennemeier and Denver 2002a; Marin et al. 2007; Malisch et al. 2010; Narayan et al. 2011a). For logistic reasons, and to minimize number of tadpoles used in the experiment, we chose the 8 h and 24 h time periods to capture short-term whole-body CORT response of tadpoles when exposed to new stressors. These time points were chosen based on existing literature on closely related species, which show that CORT levels of mid-larval stage tadpoles change several hours after stress exposure (e.g. Glennemeier and Denver 2002a; Narayan et al. 2011b).

Prior to CORT sampling, individuals were immersed in 1L of RSW with an overdose (2 g/L) of dissolved buffered MS222 (Ethyl-3-aminobenzoate-methanesulfonate, Sigma–Aldrich, E10521) until they were no longer responsive (Cakir and Strauch 2005; Ramlochansingh et al. 2014). Individuals were then placed on a paper towel to remove excess water, weighed (to the nearest 0.001 g) using a digital balance (Mettler, Type PM200), and their developmental stage recorded. Thereafter the tadpoles were placed in individually labelled PP tubes (60.549.001, Sarstedt), snap frozen in liquid nitrogen, to ensure rapid hormonal stability and death of tadpoles, and stored at − 80 °C.

CORT extraction from the frozen samples was done at Uppsala University following Burraco et al. 2015 (with minor modifications, see Mausbach et al. 2022). Individual samples were chosen haphazardly first within the 8 h and then within the 24 h blocks and allowed to thaw for 5 min. at room temperature. The tissue was homogenized for 20–30 s using a Qiagen TissueRuptor II homogenizer equipped with a metal probe, which was cleaned with 99% alcohol (EtOH) and double deionized water (ddH_2_0) between samples. 0.080–0.095 g of a given sample was then transferred to a new vial containing 1500 µl of VWR Ethyl Acetate (99.8%, Sigma–Aldrich, 270,989). The sample was then shaken for 30 s using a VWR Analog Vortex Mixer, followed by shaking for 30 min in an automatic shaker in a 4 °C room. Finally, the sample was centrifuged for 15 min at 5000 RPM, and 1450 µl Ethyl acetate supernatant was pipetted into a 2 ml Safe-lock Eppendorf Tube. The samples were stored at − 20 °C until evaporated in a speed vac at 45 °C (SpeedVac plus, SC110A attached to Savant, Gel Pump GP110). All samples were subsequently transported to the Swiss Federal Institute of Aquatic Science and Technology (EAWAG) in Switzerland and reconstituted in 115 µl assay buffer (Arbor Assays Detect X Corticosterone Enzyme Immunoassay Kit) and 5 µl 99% EtOH (Burraco et al. 2015).

The samples were analysed using Arbor Assays Detect X Corticosterone Enzyme Immunoassay Kit (K014-H1/H5). We previously confirmed that corticosterone is the main glucocorticoid in *R. arvalis* tadpoles (Mausbach et al. 2022). The samples were processed in haphazard order within the 8 h and 24 h blocks following the provided instructions for the kit, washed in a BioTek plate washer (BioTek, ELx50), and their optical density (O.D.) at 450 nm measured on a SpectraMax 190 plate reader (Molecular devices). Each sample was run in duplicates. (It was not possible to add more than two technical replicates due to small size of *R. arvalis* tadpoles). Following standard endocrinological methods (Wolfgang Goymann, personal communication), each duplicate was pipetted next to each other on a given plate (e.g. position C5 and D5). Although this comes at a cost of statistical non-independence, it minimizes plate contamination and pipetting errors.

The O.D. values from the plate reader were transformed to hormonal concentration (pg/ml) by interpolation to an automatically calculated standard curve provided by the manufacturer (https://www.myassays.com/arbor-assays-detectx-corticosterone-(od).assay). The standard curve covered a concentration range from 78 to 10,000 pg/ml. For statistical analyses, the average CORT values of the two duplicates of a given tadpole were used. The Arbor assay kit manual gives a sensitivity for the used CORT assay of 18.6 pg/ml and a detection limit of 16.9 pg/ml (Arbor Assays Detect X Corticosterone Enzyme Immunoassay Kit, K014-H1/H5) and has been tested for cross reactivities for several substances/metabolites (Arbor Assays Detect X Corticosterone Enzyme Immunoassay Kit, K014-H1/H5). Except for Desoxycorticosterone (12.3%), a metabolite of Corticosterone, cross reactivities with CORT metabolites are all below 0.8%. Plate intra- and inter-assay coefficients of variation were calculated using standards of low (standard conc. 5–8, Arbor assays) and high (standard conc. 1–4, Arbor assay) level groups and a pooled sample of non-experimental tadpoles (extra individuals) on each plate (pool only five instead of six EIA plates used in total). The pooled samples of tadpoles were of similar stage and extracted the same way as the experimental individuals. Intra-assay coefficient of variation for the two standards groups was, on average, 11.00% (low: 13.87%, high: 8.14%) and for the pooled sample 6.78%. Inter-assay coefficient of variation was, on average, 5.40% (low: 4.61%, high: 6.19%) for standard groups and 15.22% for the pool. The average coefficient of variation of duplicates run over all six plates given by the calculations of the automated program was 10.87%. The hormonal values were corrected for amount of µl of sample and mg of tadpole tissue extracted and is reported as pg/mg.

### Statistical analyses

All statistical analyses were done in R (Version 3.4.2) and RStudio (Version 1.1.463; R Core Team 2013). Any samples missing data or below developmental stage G27.5 were removed. One outlier (sample 2704) was removed due to abnormally high CORT value (5.2 times higher than the next highest value). Normality of all models was assessed visually using QQ plots and by checking the distribution of residuals. Note that family level replication (2–3 individuals/family) was too low to include family as a random effect in the statistical analysis.

Activity level (%) was analysed using repeated measures mixed models and the packages car, lme and lme4 (Bates et al. 2015; Fox and Weisberg 2019; Pinheiro et al. 2020). We first ran a full model with population, pH treatment, predator treatment, and time period (Pre-, Post 1-, and Post 2/3 exposure), as well as their two-, three-, and four-way interactions as fixed factors, and individual ID as a random effect. Activity was square root transformed to reach normality. Due to a significant four-way interaction (Pop × Predator × pH × Time), the full model was followed by type III ANOVAs within each time period separately. In these models, population, pH treatment, predator treatment, and their two- and three-way interactions were used as fixed effects. Non-significant three-way interactions were then removed sequentially, and only final models presented here. For the pre-time point, an additional ANOVA was run without predator treatment as fixed effect (as this treatment was not applied at this point) but there were no treatment effects and results are not reported further.

To analyse CORT levels (pg/mg), Type III ANOVAs using nlme (Pinheiro et al. 2020) (using: options (contrast = c(“contr.sum”,”contr.poly”))) and car (Fox and Weisberg 2019) packages were used. In the first model, population, pH treatment, predator treatment, time (8 and 24 h), and their two-, three-, and four-way interactions were used as fixed factors. Although none of the time effects were statistically significant (all *p* > 0.05), separate analyses within each time period (8 h and 24 h) were also conducted as the treatments appeared to differ within populations in a visual inspection. For CORT, one NOP individual at 8 h (Neutral pH–Predator treatment) could not be sampled, which gives a total *N* = 159 for this trait.

Many tadpole traits, including behaviour and CORT levels, can covary with body size (e.g. Glennemeier and Denver 2002b; Dahl et al. 2012). To test for the effects of individual body size on activity and CORT, analyses were also conducted within each of the two populations (AOP and NOP) with tadpole mass as a covariate. Models with body mass were conducted within each population because body mass is strongly confounded with population identity (AOP tadpoles, mean mass: 0.274 g; are substantially larger than NOP tadpoles: 0.167 g). A factorial ANOVA on developmental stage showed that there were no significant differences between populations or treatments in developmental stage at the time of sampling (Supplemental Table 5). Finally, tadpole mass or developmental stage had no significant effects on either behaviour or CORT within populations (Supplemental Tables 2 and 4).

## Results

### Behavioural responses

**Fig. 2:**
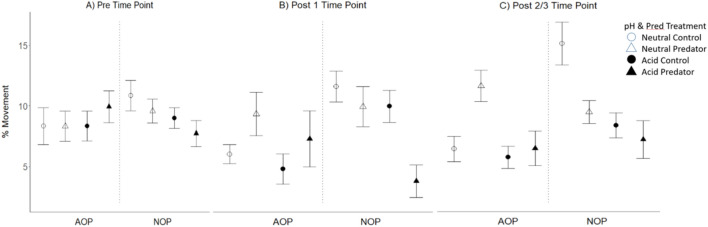
Mean ± SE of movement activity (% time moved) of individual *Rana arvalis* tadpoles before and after exposure to predator or no-predator cue. Movement % is shown for two populations (acid origin: AOP and neutral origin: NOP). The Pre- indicates a 5 min period prior to addition of predator cue treatment, Post 1 the 5 min after cue addition and Post 2/3 the final 10 min. (Total time for this behavioural recording is 20 min. See Fig. 1. *N* = 20 for each population-treatment combination

Tadpole behaviour was influenced by pH x predator x population x time interactions (Table 1, Fig. 2). Within time point analyses found that at Pre-time (i.e. after being placed in acid or neutral treatment but before addition of the predator cue) there were no significant population or treatment effects on activity of tadpoles (Fig. 2, Table 2, Supplemental Table 2). At Post1, tadpole activity was overall lower in the acid treatment (Fig. 2, Table 2) and a significant population × predator effect arose as AOP tadpoles increased*,* whereas NOP tadpoles decreased activity in the presence of predator cues relative to no-cue control (Fig. 2, Table 2). The effect in NOP tadpoles was especially strong in the acidic pH, bringing about a significant pH × predator interaction (Supplemental Table 2). At Post2/3, a significant population × pH × predator interaction revealed a difference between the populations in the response to the joint pH and predator cue treatments. This effect arose because of an increase in activity of AOP tadpoles and, in particular, a decrease in activity of NOP tadpoles in the presence of predator cue was only evident in the neutral pH treatment (Fig. 2, Table 2). Tadpoles from both populations were less active in the acidic pH also during the Post2/3 period (Supplemental Table 2).

**Table 1:** Mixed model analysis of behavioural activity (% of movement) of *Rana arvalis* tadpoles from two populations (AOP and NOP) across three time points (Pre, Post 1, and Post 2/3, Fig. 1)

Factors	*Chi*^*2*^	*df*	*P*
Population	1.39	1	*0.056*
pH treatment	15.07	1	**0.001**
Predator treatment	19.60	1	** < 0.001**
Time	16.65	2	**0.024**
Pop × pH	1.64	1	0.119
Pop × Predator	11.58	1	** < 0.001**
pH × Pred	4.69	1	0.090
Pop × Time	3.18	2	0.147
pH × Time	6.19	2	0.115
Pred × Time	10.87	2	**0.035**
Pop × pH × Pred	1.57	1	0.321
Pop × pH × Time	5.53	2	*0.052*
Pop × Pred × Time	5.55	2	0.065
pH × Pred × Time	6.45	2	**0.020**
Pop × pH × Pred × Time	8.72	2	**0.010**
Random Effect—ID	–	–	** < 0.001**

Tadpole behaviour was monitored in two pH (neutral or acid) and two predator cue (predator-cue and no-cue) treatment combinations. Tadpole identity (ID) was included as a random effect. Significant effects (*p* < 0.05) are highlighted in bold. *N* = 160

**Table 2:** Linear models of behavioural activity (% movement) of *Rana arvalis* tadpoles from two populations (AOP and NOP) within three time points (Pre, Post 1, and Post 2/3)

Factors	Pre				Post 1				Post 2/3			
	*SS*	*df*	*F*	*P*	*SS*	*Df*	*F*	*p*	*SS*	*df*	*F*	*P*
Population	1.06	1	1.12	0.291	0.49	1	0.29	0.591	0.50	1	0.46	0.497
pH treatment	0.97	1	1.03	0.312	16.64	1	9.86	**0.002**	3.07	1	2.86	0.094
Predator treatment	0.52	1	0.55	0.459	22.49	1	13.31	** < 0.001**	1.74	1	1.61	0.206
Pop × pH treatment	1.94	1	2.06	0.153	0.36	1	0.22	0.643	1.33	1	1.24	0.268
Pop × Pred treatment	2.59	1	2.76	0.100	14.61	1	8.65	**0.004**	0.55	1	0.51	0.478
pH × Pred treatment	0.13	1	0.14	0.709	3.88	1	2.30	0.132	0.75	1	0.70	0.405
Pop × pH × Pred									4.73	1	4.39	**0.038**
Residual	140.15	149			251.72	149			159.55	148		

**Fig. 3:**
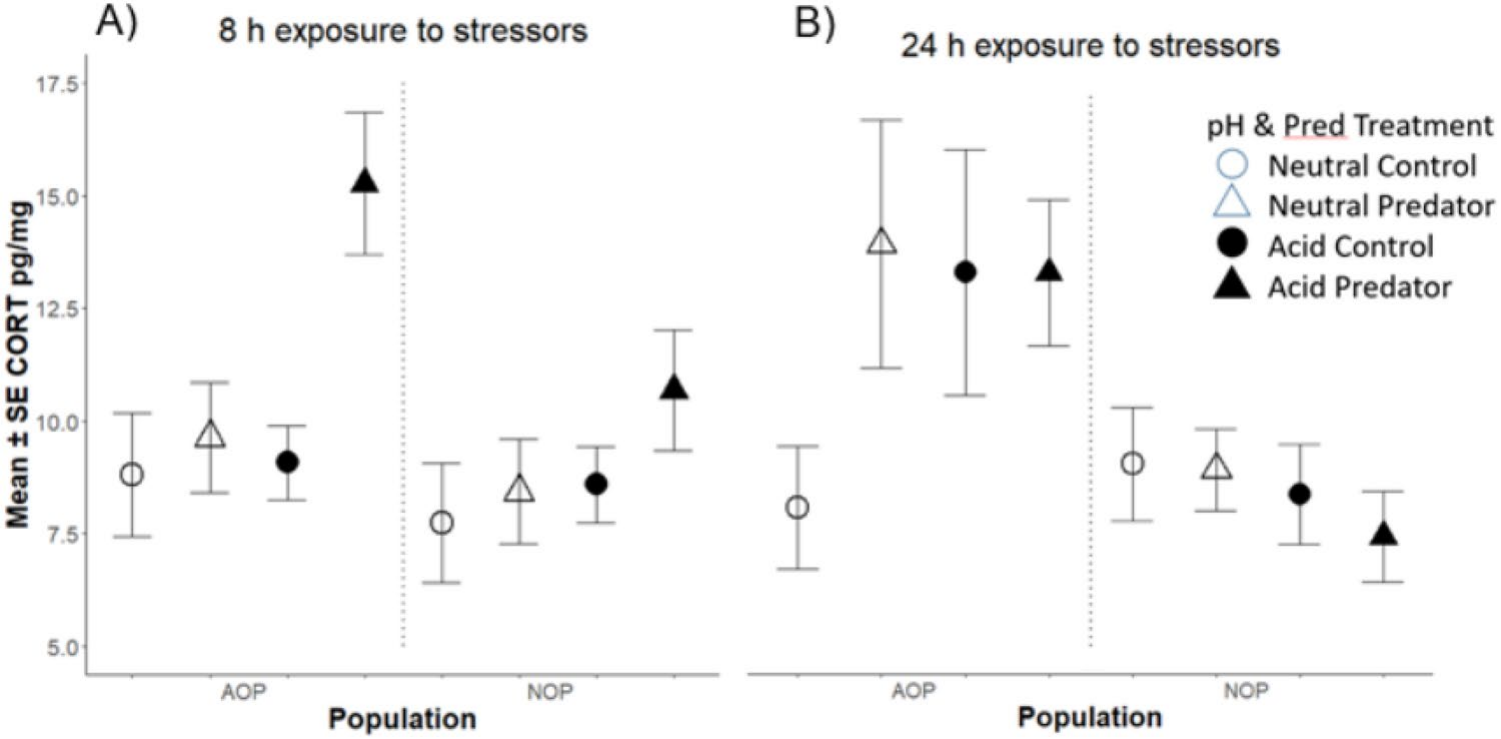
Mean ± SE of body Corticosterone (CORT) of *Rana arvalis* tadpoles from two populations (AOP and NOP) after exposure to a combination of two pH (acid and neutral) and two predator cue (predator cue and no-cue) treatments for either **A** 8 h or **B** 24 h. Treatment combinations indicated are neutral pH–no predator cue (open circle), neutral pH–predator cue (solid circle), acid pH–no predator cue (open triangle), acid pH–predator cue (solid triangle). *N* = 10 for each population–treatment–time combination, except *N* = 9 for NOP in the neutral pH–predator treatment at 8 h

Tadpole behaviour was monitored in two pH (neutral or acid) and two predator cue (predator cue and no-cue) treatment combinations. Significant effects are highlighted in bold. For sample size (*N*) and population-treatment means see Fig. 3

### Corticosterone levels

In the full model, populations differed significantly in CORT expression, but there were no significant treatment or time effects (Supplemental Table 3). Because visual inspection (Fig. 3) indicated that the population main effects were influenced by time dependent treatment effects, we next analysed the data within each time period. At 8 h, there were significant population, pH and predator effects (Table 3, Fig. 3). Specifically, CORT levels of AOP tadpoles were elevated in the acid-predator cue treatment combination relative to the other three treatment combinations (Fig. 3A, Supplemental Table 4). Visually, there was an increase in CORT for the acid-predator cue treatment combination also in NOP, but this effect was not statistically significant (Fig. 3A). At 24 h, the population main effect was retained, but none of the treatment effects were significant (Table 3). The population main effect at 24 h was driven by the AOP tadpoles having, on average, substantially elevated CORT levels in all three stress treatments (acid–no predator, neutral–predator, and acid–predator; Table 3, Fig. 3), whereas NOP tadpoles did not show CORT responses in any of the three stress treatments (Table 3, Fig. 3). The two populations had comparable CORT levels in the neutral pH–no-cue treatment.

**Table 3:** Linear models of corticosterone variation in *Rana arvalis* tadpoles originating from two populations (AOP and NOP) and exposed to two pH (neutral or acid) and two predator cue (predator cue and no-cue) treatment combinations for either 8 h or 24 h

Factors	8 h				24 h			
	*SS*	*Df*	*F*	*P*	*SS*	*df*	*F*	*P*
Population	86.60	1	5.83	**0.018**	343.32	1	11.32	**0.013**
pH treatment	68.02	1	4.58	**0.035**	50.85	1	1.68	0.200
Predator treatment	61.68	1	4.16	**0.045**	31.8	1	1.05	0.309
Pop × pH treat	9.51	1	0.64	0.426	58.4	1	1.92	0.170
Pop × Pred. treat	22.80	1	1.54	0.219	56.18	1	1.85	0.175
pH × Pred. treat	57.20	1	3.85	*0.053*	57.05	1	1.88	0.178
Residuals	1053.80	71			2123.88	70		

Significant effects (*P* < 0.05) are highlighted in bold

The Population × pH × Predator effect was not significant and was removed from these final models

## Discussion

We found differences between two *R. arvalis* populations in short-term stress induced behavioural activity and CORT expression: tadpoles from an acid origin population (AOP) had a qualitatively different behavioural response to predator cues and were generally more responsive in CORT levels than tadpoles from a neutral pH origin population (NOP). These findings indicate genotype–environment interactions in behavioural and hormonal responses, which likely reflect the different acidity-mediated selective histories of these populations (Hangartner et al. 2011; Egea-Serrano et al. 2014).

### Behavioural responses

In terms of short-term predator cues, NOP tadpoles exposed to predator cue *reduced*, while AOP tadpoles *increased* their activity (as compared to tadpoles in the no-cue treatments). A reduction in activity level, as shown by the NOP tadpoles, is one of the most ubiquitous behavioural responses of tadpoles to predator presence that reduces the likelihood of detection by the predator (e.g. Lima and Dill 1990; Kats and Dill 1998; Ferrari et al. 2010), and has been shown also in *R. arvalis* (Laurila et al. 2006; Egea-Serrano et al. 2014). Increased activity, as shown by the AOP tadpoles, however, is a more uncommon response and may reflect active escape from predators (e.g. Brown et al. 2019). However, we also saw an apparent weakening of the behavioural response in acidic pH over time (Post 1 vs. Post 2/3, Fig. 3), which suggests that tadpoles acclimate to the presence of the cue, that acid water may impair the chemosensory system with delay, or that tadpoles first increase (escape response) but then decrease activity (hiding).

We suggest that the two populations may differ in their predator avoidance strategies (i.e. by avoid detection by hiding versus actively swimming away). Interestingly, in a previous study we found that both NOP and AOP tadpoles *reduced* activity under chronic exposure to predator cues (Egea-Serrano et al. 2014). However, our current experiment differed in several aspects in rearing and experimentation from Egea-Serrano et al. (2014): from rearing singly (here) *vs.* in groups, and including only chemical cues (here) vs. chemical and visual cues, to short-term (here) vs. chronic exposure to predator cues. The qualitatively different responses in the present study may hence reflect context dependency in predator avoidance. Importantly, the former study found that AOP tadpoles were generally more active than NOP tadpoles, yet had higher survival when exposed to free-ranging predators (Egea-Serrano et al. 2014). This suggests stronger adaptation in AOP to predation, and it is possible that the rapid behavioural evasion (here), deeper tails (Egea-Serrano et al. 2014) and deeper tail muscles (Mausbach et al. 2022) are all adaptations to elevated predation risk in acidic environments. One explanation for lack of adaptation in NOP tadpoles might be that being adapted to acid and predator stress (AOP tadpoles) compromises other life-history traits. In terms of fitness consequences, the most obvious trade-off, based on studies thus far, is that AOP tadpoles develop slower than NOP tadpoles in controlled conditions—indicating genetic divergence in life-history traits (slow development is potentially costly in seasonal populations at northern latitudes, discussed for example in Hangartner et al. 2011). In addition, AOP produce fewer but larger eggs (Räsänen et al. 2008).

Due to differences between our study populations in tadpole developmental rates (AOP develop slower, Hangartner et al. 2011; Mausbach et al. 2022), the behavioural measurements were carried out first with NOP individuals and then with AOP two days later. All efforts were made to negate biases in both data collection and analysis: the experiment was conducted under similar conditions, with the same person (NS) conducting the experiment to prevent changes in methodology, and all videos were analyzed blind and in a random order to prevent bias in methodology or interpretation.

### Corticosterone expression

In terms of tissue CORT levels, AOP tadpoles were more hormonally responsive than NOP tadpoles: they had increased CORT levels after 8 h exposure in the acid-predator cue treatment as well as after 24 h in all three ‘stress’ treatments (acid and/or predator cue). In contrast, there were no statistically significant stress responses in CORT for NOP tadpoles at either 8 h or at 24 h. These differences between the populations in hormonal responses, as those in behaviour, indicate divergent phenotypic plasticity in stress responses.

Different CORT responses have been observed under short-term and chronic stress in a range of taxa (e.g. reviewed in Sapolsky et al. 2000; Kitaysky et al. 2003; Sheriff et al. 2009; Vitousek et al. 2019; Gormally and Romero 2020). However, few studies have compared CORT expression among populations (Mausbach et al. 2022) and in responses to predators in presence of other stressors. Yet these are important aspects of stress responses in the wild, as populations vary in selective histories, organisms typically face several stressors at once, and predator responses are often context dependent (e.g. Teplitsky et al. 2007; Egea-Serrano et al. 2014; Groner et al. 2014; Relyea et al. 2018). In terms of predation risk, previous studies have found both increased and decreased CORT expression. For instance, *Pelobates cultripes* tadpoles lowered their CORT levels when exposed to native predators, but not when exposed to invasive predators (Burraco and Gomez-Mestre 2016). Other studies have found that CORT responses to predators can be both time- and population-specific (e.g. Dahl et al. 2012; Middlemis Maher et al. 2013).

As indicated by our study here, AOP tadpoles may have a higher ability to increase CORT expression after an acute stressor, which further may facilitate an adaptive fast-escape response. As invertebrate predator densities generally are lower in sites with neutral pH, and acid origin populations seem better adapted to predators along our study gradient (Hangartner et al. 2011, 2012a; Egea-Serrano et al. 2014), we did expect divergent CORT responses in these divergent populations. Along these lines, a previous study on *R. temporaria* tadpoles showed higher CORT expression in populations with higher predator densities along a latitudinal gradient (Dahl et al. 2012). Whilst AOP tadpoles in our study seem to be more responsive and better adapted to acid and predator stress, a possibility for the non-significant CORT response of NOP tadpoles to predator cue would be their naivete or inability to sense predators under acidic conditions. However, given the observed reduction of NOP tadpoles in behavioural activity in the acid-predator cue treatment, this is unlikely. Further studies are clearly needed to assess drivers of these population differences.

A couple of caveats need to be considered for assessment of CORT levels in our study. First, measurements of CORT from tadpole tissue (here: whole-body CORT) are integrative but may not accurately reflect the immediate hormonal stress response, as would be the case if plasma CORT was measured within minutes (e.g. De Kloet et al. 2005; Burraco et al. 2015; Gormally and Romero 2020). It would have been particularly interesting to compare immediate plasma CORT levels with the immediate behavioural response, but this was prohibited because of the small size of *R. arvalis* tadpoles (non-lethal sampling is not possible and plasma levels are too low for reliable individual level CORT assessment). Second, the apparent weaker CORT response of NOP tadpoles could also arise if our measuring time points did not capture temporal variation in CORT responses (e.g. Glennemeier and Denver 2002a; Gormally and Romero 2020). For example, in one study *Lithobates sylvatica* tadpoles exposed to predator stress showed reduced CORT levels after 4 h, but increased CORT levels after 4 days when a whole-body corticosterone analysis was performed (Middlemis Maher et al. 2013), whereas in another study also using whole-body corticosterone *L. sylvatica* showed increased CORT levels already 10–20 min after exposure to acute predator stress (Bennett et al. 2016). Moreover, as both circadian rhythm and tadpole developmental stage can strongly influence CORT levels (e.g. Pancak and Taylor 1983; Mausbach et al. 2022), it was necessary to sample within a similar time window (not spread out sampling over many days) to reduce variability. As CORT responses are very context dependent (e.g. Schoenle et al. 2018), and different populations may react in different time windows (although little studied to date), it is possible that we missed the time of peak increase in CORT levels in NOP (i.e. only captured a time of increasing, or decreasing levels). However, the observed elevated CORT levels in tissue within 8–24 h could reflect allocation of energy to competing stress responses—such as behavioural versus morphological defenses (Hayes and Wu 1994; Denver 2009).

### Inferences from stress induced behaviour *versus* physiology

Immediate behavioural (first 15–20 min) and short-term (8 and 24 h) integrated physiological stress responses can be informative of different organismal stress responses (Gormally and Romero 2020). In our study, behavioural responses showed that tadpoles from both populations were able to detect predator cues even in acidic pH, whereas hormonal results would have suggested that only AOP tadpoles react to predator or acid stress. The ability to respond to predators under acidic conditions is in line with former studies on *P. cultripes* (Burraco & Gomez-Mestre 2016; Florencio et al. 2020) and *R. arvalis* (Egea-Serrano et al. 2014) and, jointly with previous studies, indicates that unlike many marine and freshwater fish (Leduc et al. 2013), the olfactory system of amphibian larvae may not be impaired by acidic pH. In terms of effects of acidity on stress responses, previous studies have found varying responses. These range from reduced behavioural activity (e.g. Egea-Serrano et al. 2014) to increased CORT levels under chronic exposure (several weeks) to acidic pH (anurans: Burraco & Gomez-Mestre 2016; Florencio et al. 2020; salamanders: Chambers et al. 2013). For example, Woodley (2014) found that while *Desmognathus ochrophaeus* salamanders exposed to acidic pH did reduce their activity levels, there was no effect of pH on CORT levels. On the other hand, *P. cultripes* tadpoles showed decreased CORT levels when exposed to predator stress and increased levels in acidic water, but no interactive effect of predator cue and pH (Florencio et al. 2020). Such variation in interactive effects of predator–pH stress reflects high context dependency of stress responses and could have several reasons. These include differences in measured endpoints (plasma versus tissue CORT) and length and type of exposure (hours versus weeks, predator chemical cues only versus chemical and visual cues), as well as differences between species and populations. Combining the information from current study on short-term responses, with our study on the chronic CORT levels and tadpole morphology (Mausbach et al. 2022), and chronic pH and predator stress exposures (Egea-Serrano et al. 2014), suggests that whilst AOP are more responsive (in short-term CORT measures) they have lower baseline CORT and constitutively higher activity levels, but deeper tail muscles as well as higher growth rate but slower development rates. Furthermore, the results from Mausbach et al. 2022 indicate that the lower CORT levels of AOP tadpoles are statistically associated with deeper tail muscles and slower development (Mausbach et al. 2022). Hence this indicates that CORT is linked to many other traits and might be directly involved in divergence of populations. Taken together, these differential responses highlight the need for more integrative studies on organismal stress responses, including variation among populations.

## Conclusions

Our study sheds light on phenotypic plasticity and adaptation in organismal stress responses. The differences between our two study populations in behavioural and hormonal stress responses support adaptive divergence of *R. arvalis* populations along an environmental stress gradient (Egea-Serrano et al. 2014). While the fitness consequences of these divergent plastic responses remain to be directly tested, our results suggest that differences in selective history (here via acidity and predators) can lead to divergent selection on behavioural and physiological plasticity (Ghalambor et al. 2007). Such differences may have implications for the ability of populations to respond to environmental change (Ghalambor et al. 2007; Merilä and Hendry 2014; Fox et al. 2019). Some caution is warranted, however, as we compared only one AOP with one NOP. Whilst pH and predators have been found to be the main driver of divergence among our study populations in larval growth and morphology (e.g. Egea-Serrano et al. 2014; Hangartner et al. 2012a, b), and is associated with divergence above neutral expectations (Hangartner et al. 2011), a variety of other biotic and abiotic factors (incl. latitude) could also influence the responses of the tadpoles. Further studies on larger number of populations are needed for testing the generality of the observed patterns.

Our experiment was conducted on individually reared tadpoles in simple laboratory conditions and—given the high context dependency of hormonal and behavioural responses—there is clearly a need for studies in different ecologically relevant settings. For example, experiments using multiple interacting individuals, larger arenas and different predator cues (e.g. visual cues in combination with chemical cues; Hettyey et al. 2012; Egea-Serrano et al. 2014), and investigating the temporal sensitivity of predator responses, would shed additional light on how antipredator strategies of the populations differ. While more work is needed to create a better understanding of how abiotic and biotic stressors affect organisms, our findings highlight the need to study among population divergence in physiological and behavioural plasticity under stress interactions. Such insight would inform us about mechanisms of adaptation in nature and the role of plasticity in stress responses, and inform the use of behavioural and hormonal responses as bioindicators of stress (Narayan et al. 2019; Gormally and Romero 2020).

## Supplementary Information

Below is the link to the electronic supplementary material.

Supplementary file1 (XLSX 48 KB)

Supplementary file2 (DOCX 27 KB)

## Data Availability

The datasets generated and/or analysed during the current study are available upon publication at Dryad, persistent web link to data sets will be provided.

## Code availability

Codes are available upon request.
