# Supplementary material for: Short-term responses of Rana arvalis tadpoles to pH and predator stress: adaptive divergence in behavioural and physiological plasticity?
Source: J Comp Physiol B. 2022 Jul 20;192(5):669–82. doi: 10.1007/s00360-022-01449-2 (PMC9388420; doi:10.1007/s00360-022-01449-2)
Supplement: Supplementary file 2 — Supplementary file2 (DOCX 27 KB) [file 360_2022_1449_MOESM2_ESM.docx]

**Supplemental Tables**

**Supplemental Table 1.** ICC1 analysis of 20 randomly selected videos comparing auto- tracking to manual movement counts. Significant values are shown in bold.

|  | *ICC* | *Upper Bound* | *F* | *df1* | *df2* | *P* |
| --- | --- | --- | --- | --- | --- | --- |
| ICC 1 | 0.83 | 0.92 | 11 | 19 | 20 | **<0.001** |
| ICC 1k | 0.90 | 0.96 | 11 | 19 | 20 | **<0.001** |

**Supplemental Table 2.** Analysis of variance of behavioural activity (% movement) of *Rana arvalis* tadpoles from a A) neutral origin population (NOP) and B) acid origin population (AOP) within three time points (Pre: prior to predator cue addition, Post 1 and Post 2/3 after predator cue additions, see Figure 1 for details). Tadpole weight and stage as covariate was removed due to non- significance. Significant effects (P < 0.05) are highlighted in bold.

|  | Pre | | | | Post1 | | | | Post2/3 | | | |
| --- | --- | --- | --- | --- | --- | --- | --- | --- | --- | --- | --- | --- |
| A) AOP | | | | | | | | | | | | |
| *Factors* | *SS* | *df* | *F* | *p* | *SS* | *df* | *F* | *p* | *SS* | *df* | *F* | *p* |
| pH | 0.82 | 1 | 0.66 | 0.419 | 5.44 | 1 | 2.53 | 0.116 | 11.94 | 1 | 10.24 | **0.002** |
| Pred | 1.88 | 1 | 1.51 | 0.223 | 0.17 | 1 | 0.08 | 0.777 | 0.08 | 1 | 0.07 | 0.800 |
| pH×Pred | 0.60 | 1 | 0.49 | 0.488 | 0.22 | 1 | 0.10 | 0.752 | 4.97 | 1 | 4.26 | **0.042** |
| Residual | 94.49 | 76 |  |  | 163.479 | 76 |  |  | 88.61 | 76 |  |  |
| B) NOP | | | | | | | | | | | | |
| *Factors* | *SS* | *df* | *F* | *p* | *SS* | *df* | *F* | *p* | *SS* | *df* | *F* | *P* |
| pH | 1.50 | 1 | 2.39 | 0.127 | 17.68 | 1 | 14.73 | **<0.001** | 3.07 | 1 | 3.12 | 0.080 |
| Predator | 0.98 | 1 | 1.57 | 0.214 | 23.00 | 1 | 19.159 | **<0.001** | 1.74 | 1 | 1.77 | 0.188 |
| pH×Pred | 0.08 | 1 | 0.12 | 0.726 | 5.50 | 1 | 4.59 | **0.036** | 0.75 | 1 | 0.76 | 0.385 |
| Residual | 45.11 | 72 |  |  | 86.397 | 72 |  |  | 70.94 | 72 |  |  |

**Supplemental Table 3.** Analysis of variance on corticosterone (CORT) levels of *Rana arvalis* tadpoles from two populations (AOP and NOP) after exposure to the combination of two pH (neutral and acid) and two predator cue (predator cue and no-cue) treatments for either 8 or 24h (time). All four and three-way interactions were not significant and were removed from these final models. Significant effects (P < 0.05) are highlighted in bold.

| *Factors* | *SS* | *df* | *F* | *p* |
| --- | --- | --- | --- | --- |
| Population | 1002.2 | 1 | 5.86 | **0.017** |
| pH treatment | 190.7 | 1 | 1.12 | 0.293 |
| Predator treatment | 0.1 | 1 | 0.00 | 0.980 |
| Time | 27.9 | 1 | 0.69 | 0.687 |
| Pop×pH | 17.4 | 1 | 0.10 | 0.751 |
| Pop×Pred | 407.3 | 1 | 2.38 | 0.125 |
| pH×Pred | 147.4 | 1 | 0.86 | 0.355 |
| Pop×Time | 309.3 | 1 | 1.81 | 0.181 |
| pH×Time | 291.2 | 1 | 1.70 | 0.194 |
| Pred×Time | 66.6 | 1 | 0.39 | 0.534 |
| Residuals | 24806.8 | 145 |  |  |

**Supplemental Table 4.** Analysis of variance on CORT expression of *Rana arvali*s tadpoles from A) NOP and B) AOP population 8h or 24h after exposure to a combination of two pH (acid or neutral) and two predator cue (predator cue or no-cue) treatments. Tadpole weight and stage were included as a covariate but had no significant effects, and were removed from the model. Significant effects (P < 0.05) highlighted in bold.

|  | *8 h* | | | | *24 h* | | | |
| --- | --- | --- | --- | --- | --- | --- | --- | --- |
| A) AOP | | | | | | | | |
| *Factors* | *SS* | *df* | *F* | *p* | *SS* | *df* | *F* | *p* |
| pH treatment | 158.590 | 1 | 9.841 | **0.003** | 1.99 | 1 | 0.041 | 0.841 |
| Predator treat. | 191.37 | 1 | 11.875 | **0.001** | 0.00 | 1 | 0.00 | 0.996 |
| pH×Pred | 71.88 | 1 | 4.604 | **0.042** | 87.24 | 1 | 1.800 | 0.188 |
| Residuals | 580.14 | 36 |  |  | 1696.46 | 35 |  |  |
| B) NOP | | | | | | | | |
| *Factors* | *SS* | *df* | *F* | *p* | *SS* | *df* | *F* | *p* |
| pH treatment | 23.89 | 1 | 1.78 | 0.190 | 11.03 | 1 | 0.948 | 0.337 |
| Predator treat. | 21.80 | 1 | 1.631 | 0.210 | 4.63 | 1 | 0.398 | 0.532 |
| pH×Pred | 4.55 | 1 | 0.340 | 0.564 | 1.66 | 1 | 0.143 | 0.708 |
| Residuals | 454.43 | 34 |  |  | 395.57 | 34 |  |  |

**Supplemental Table 5.** Factorial analysis of variance on developmental stage variation in *Rana arvalis* tadpoles exposed to a combination of two pH (acid or neutral) and two predator cue (predator cue or no-cue) treatments. All three-way interactions were non-significant, and thus removed.

| *Factors* | *SS* | *df* | *F* | *p* |
| --- | --- | --- | --- | --- |
| Population | 3.4 | 1 | 3.250 | 0.073 |
| pH treatment | 0.1 | 1 | 0.052 | 0.809 |
| Predator treat. | 0.9 | 1 | 0.843 | 0.360 |
| Pred x Pop | 2.4 | 1 | 2.260 | 0.135 |
| Pop x pH | 0.1 | 1 | 0.124 | 0.725 |
| Pred x pH | 0.2 | 1 | 0.191 | 0.662 |
| Residuals | 158.6 | 150 |  |  |
